# Spectacle Independence After Cataract Surgery: A Prospective Study With a Multifocal Intraocular Lens

**Published:** 2020-01-01

**Authors:** German Roberto Bianchi

**Affiliations:** Clínica de Ojos Dr. Nano. Centro Panamericana. Blas Parera 4201. B1636CSS - OLIVOS. Buenos Aires, Argentina.

**Keywords:** Presbyopia, Pseudophakic Intraocular Lens, Multifocal Intraocular Lens, Refractive Surgery, Cataract Surgery

## Abstract

Our aim was to review clinical outcome and patient satisfaction after cataract surgery to obtain spectacle independence following multifocal intraocular lens (IOL) implantation. A prospective case-series study was designed to evaluate the safety and efficacy of the Hanita FullRange pseudophakic multifocal intraocular lens in patients with programmed cataract surgery, performed between October 2017 and May 2018, with follow-up after 12 months. Manifest refraction spherical equivalent (SE), SE refractive accuracy, uncorrected distance (UDVA), intermediate (UIVA) and near visual acuity (UNVA) and a binocular defocus curve were evaluated. In addition, a short “satisfaction questionnaire” was developed. Surgeries were performed without viscoelastic substance. The corneal endothelial cell density (ECD), central corneal thickness (CCT) and intraocular pressure (IOP) were also evaluated. A total of 480 eyes of 240 patients with mean ± standard deviation (SD) of age of 75 ± 6.12 years were included. The mean ± standard deviation (SD) of preoperative SE was 2.0 ± 2.18 D (range; -5.50 to 4.75) which decreased to -0.04 ± 0.28 D (range; -0.75 to 0.625) 12 months after surgery. Regarding SE refractive accuracy 82.9 % of eyes obtained SE values between -0.5 and 0.5 D. There was no loss of lines of vision and 98.3% of patients achieved UDVA between 20/20 and 20/25. The UNVA (binocular) obtained was J1 for 72.5% and J2 for 27.5% of patients. Regarding defocus curve, 0.04 logMAR for -3.0 D, 0.09 logMAR for -1.5 D and 0.03 logMAR for 0 D was achieved. The mean CCT was increased by 6.62 ± 2.79 micrometer (1.24%), the mean ECD was decreased by 226.08 ± 11.63 cell/mm^2 ^(9.00 %) and the IOP remained stable one year after surgery. In response to the satisfaction questionnaire, 92% of patients stated that they had obtained spectacle independence. Finally, spectacle independence was achieved in most of the cases, with a high level of patient satisfaction one year after implantation of a FullRange IOL. No complications were detected. We concluded that the refractive efficacy of FullRange multifocal IOL was proved in majority of cases. A large follow up period is necessary in future studies to confirm the results.

## INTRODUCTION

Worldwide, life expectancy has extended and people are still active in their 60s to 80s years. They need their visual aptitude to continue, so more people seek cataract surgery [1-4]. For this reason, patients’ expectations after cataract surgery are increasing [5-7]. Moreover, patients want to obtain good uncorrected distance visual acuity and achieve good intermediate and near sight as well [4, 6-8]

Different strategies have been implemented to achieve spectacle independence after cataract surgery, and there are many options regarding intraocular lenses (IOLs) [8-15]. Monofocal IOLs are used for monovision surgical approaches and multifocal IOL platforms are improving, giving a better possibility to improve vision at all distances, but some problems have been reported related to decrease in contrast sensitivity and photopic symptoms at night (halos) [9, 10, 12-14].

The first clinical evaluation Phase 2 report from SeeLens MF (Hanita Lenses) was officially presented in June 2012 [16], Thereafter various studies were published [17-19]. The FullRange (Hanita Lenses) is the brand name of a multifocal IOL with a diffractive surface, apodized and aspheric, developed with the SeeLens MF platform. Although, some studies presented at meetings and conferences did not explore a large number of consecutive cases [17-19]. 

The purpose of this study was to evaluate safety and efficacy of this lens to obtain spectacle independence and patient satisfaction.

## Material and Methods

A prospective non-randomized case-series study was designed to evaluate safety and efficacy of the FullRange pseudophakic intraocular lens in patients with programmed cataract surgery, performed between October 2017 and May 2018, with follow-up of 12 months. The study protocol and researchers adhered to the tenets of the Declaration of Helsinki. An ethical approval was obtained from Dr. Nano Eye Clinic Institutional Review Board/Ethics Committee. Patients were informed about the study characteristics and the risks of the surgical procedure. A written informed consent was obtained prior to participation.

Patients with cataracts classified as nuclear opalescence (NO)1- nuclear color (NC)1 to NO4-NC4, according to the Lens Opacities Classification System III (LOCS III) [20], with indication for cataract surgery for both eyes, who had given their written informed consent, were included. Patients with cataracts classified as NO5-NC5 or NO6-NC6, those with post-traumatic cataracts or preoperative endothelial cell density count (ECD) below 2,000 cell/mm^2^, those with corneal pathology (herpes infection, corneal scar, previous corneal refractive surgery, moderate to severe dry eye), those with pseudoexfoliation, pupil synechiae or small pupil, uveitis, and/or previous vitreoretinal surgeries and/or previous glaucoma surgery and patients with intraoperative posterior capsular rupture with vitreous loss were excluded. Furthermore, patients with intraocular pressure (IOP) higher than 21 mmHg were excluded and another surgical technique more appropriate for them was recommended.

At baseline, all patients underwent a complete ophthalmic examination including macular ocular coherence tomography (OCT). Also, population information regarding age and gender was registered. Ocular surface disease was evaluated to rule out patients with dry eye (using vital dyes, tear break-up time and the Schirmer test). The Pentacam imaging system (Oculus, Wetzlar, Germany) was used for preoperative evaluation of the cornea (to detect regular versus irregular astigmatism). The IOL power calculation was determined using the IOL-Master equipment, with SRK/T, Haigis and Holladay formulas, accordingly the axial length of the eye [21]. The target was emmetropia in the dominant eye and -0.25 D in the non-dominant eye. Manifest refraction spherical equivalent (SE) was evaluated before and 12 months after surgery, and SE refractive accuracy was also evaluated.

The postoperative uncorrected distance visual acuity (UDVA) on the Snellen chart, the uncorrected near visual acuity (UNVA) on the Jaeger chart, and a binocular defocus curve were evaluated at last visit, 12 months after surgery. The logarithm of the minimum angle of resolution (logMAR) was calculated to obtain the defocus curve with additions from -4.0 to +2.0 D. The uncorrected intermediate visual acuity (UIVA) was evaluated by the ability to see a computer screen at 70 cm.

Surgical complications were evaluated by slit lamp, as IOL decentration or posterior capsular opacification (PCO), 12 months after surgery. A short and simple “satisfaction questionnaire” was developed for this study. Patients were asked to respond to it anonymously, alone in their homes, one year after surgery. Only 3 questions were asked including whether spectacle independence had been obtained, the preoperative surgical expectation had been achieved and if halos were experienced.

The corneal ECD and CCT were registered preoperatively, and 6 and 12 months postoperatively, using an electronic specular microscope (TOMEY EM4000). IOP was evaluated at baseline, day 1, month 1 and month 12 after surgery, using Goldmann tonometry.

FullRange characteristics (obtained from the official brochure) [22]: The FullRange MF (Hanita Lenses, Israel) is an acrylic hydrophilic (HEMA/CEOMEA) aspheric apodized diffractive multifocal IOL, with ultraviolet filter (UV-blocking) and violet light filtering chromophore. It is a foldable single-piece IOL, with the same platform as SeeLens AF (Hanita Lenses), with an optic diameter of 6.0 mm and an overall length of 13.0 mm, with a 360° continuous square edge optic (to minimize posterior capsular opacification). It is designed to be implanted from a 1.8 mm incision. Smooth diffractive steps are localized in the 4.0 mm central zone, suiting pupil sizes in different lighting conditions. The near vision add of this lens is +3.00 D greater than the distance power, equivalent to +2.4 D at the spectacle plane. Its haptics are designed with an open C-loop, with a 5° haptic angulation, increasing their stability in the capsular bag or sulcus, and decreasing the potential refractive effect that could occur with postoperative capsular contraction. Moreover, their haptic design offers a better tilt and decenter tolerance.

Both eyes were operated (with one week between surgeries) and all surgeries were performed by the same surgeon. The use of viscoelastic substances was completely avoided (the anterior chamber was maintained with an infusion/irrigation cannula, with a balanced salt solution), as in the previous publication [23]. In this study, INFINITI phacoemulsification equipment (Alcon, Forth Worth, the USA) with “OZil burst” mode (parameters: 60 limit; 70 on ms, 300 vacuum and 300 rate) was used. Vertical or horizontal “phacochop” was performed according to the cataract hardness. The IOL cartridge was introduced through a 2.8 mm corneal incision, and the IOL was placed, using the cannula to help during the unfolding process to obtain the correct IOL position in the capsular bag. Finally, an intracameral antibiotic (cefuroxime) was injected and the operation was concluded. Pre- and postoperative topical treatment was the same for all cases, starting three days before the surgery with gatifloxacin 0.5% (POEN Laboratorio, Argentina) and bromfenac 0.09% (POEN Laboratorio, Argentina), four times daily. Patients continued the treatment after surgery, adding one more drop, four times daily, as well as difluprednate 0.05% (POEN Laboratorio, Argentina). Administration of all the drops was maintained for one week. Thereafter the treatment changed to gatifloxacin 0.03% and dexamethasone 0.1% (POEN Laboratorio, Argentina), four times daily for the next 3 weeks duration.

After the objective of the study and the usual potential cataract surgery complications had been explained to the patient, extra time was taken to talk about what could be expected from surgery, since patient satisfaction after multifocal IOL implantation procedures may be partly associated with how well the surgeon explains them preoperatively. For this purpose, using easy-to-understand terminology, problems related to refractive change due to wound healing issues, inaccurate refractive results and posterior capsular opacification were explained. Also, patients were advised about potential dysphotopsia symptoms, such as halos and glare, which could be experienced at night. Moreover, they were informed that spectacle independence might not necessarily happen and some might still need spectacles for some activities or even permanently. But, as the surgeon explains, with a positive attitude, spectacle independence could presumably be achieved.

Descriptive statistical results were presented as mean, standard deviation (SD) and range. Normality of data was checked using the Kolmogorov-Smirnov test. To compare the differences between mean ECD, CCT and IOP, ANOVA (single factor) was used. A statistically significant result was considered with a *p-*value of less than 0.05. Statistical analysis was performed with the XLMiner Analysis ToolPak software (Frontline Systems Inc.). Data has been registered at “Clínica de Ojos Dr. Nano” and is available upon request to the corresponding author.

## RESULTS

From 489 surgeries, a total of 480 eyes of 240 patients with mean ± SD of age of 75 ± 6.12 years (67–82) were included (9 eyes of 9 patients were excluded because of posterior capsular rupture; in these cases, the surgery continued without problems, but a monofocal lens was implanted). The ratio of female to male was 112/128. All the operations were performed without intraoperative complications and 12 months after surgery capsular opacification did not develop in any case. In all cases, IOL was centered correctively.

**Figure 1:**
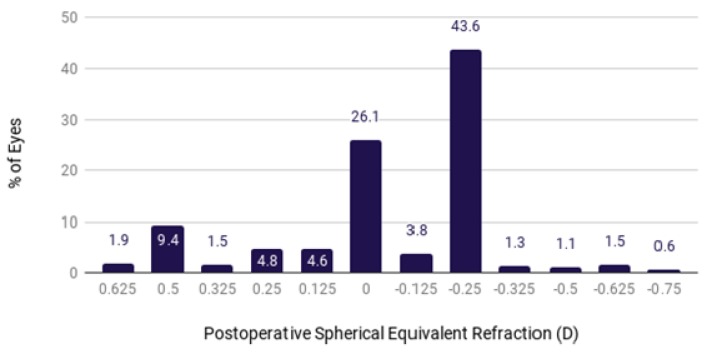
Spherical Equivalent Refraction Accuracy From FullRange Multifocal Intraocular Lens. =480 eyes, 12 months after surrgery.

Abbreviations: n: number; %: percentage; D: diopter.

**Figure 2:**
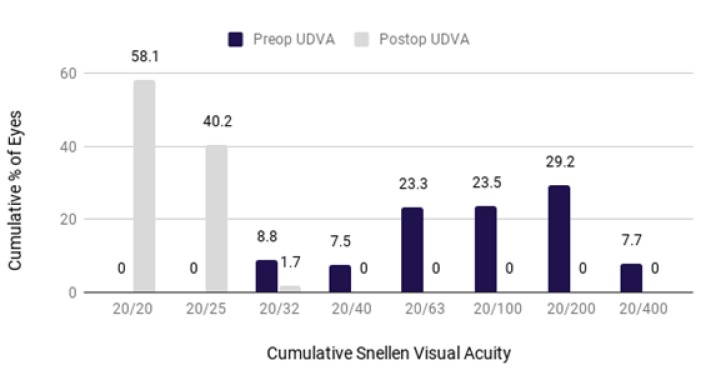
Uncorrected Distance Visual Acuity (UDVA); Preoperative Versus Postoperative Cumulative Percentage of Eyes. n=480 eyes, 12 months after surrgery. Abbreviations: n: number; %: percentage.

**Figure 3:**
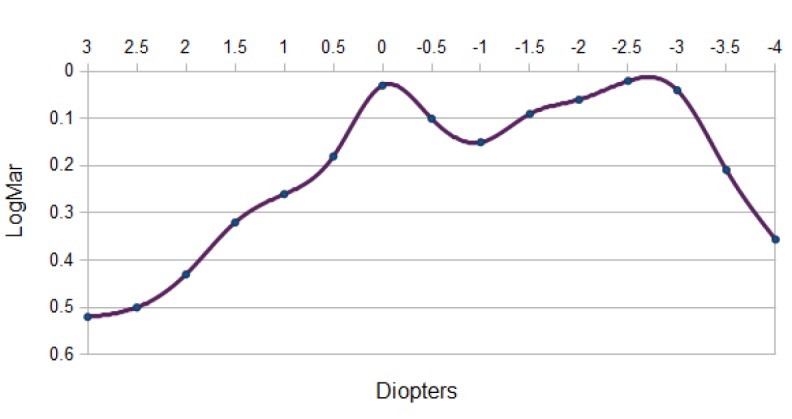
Binocular defocus curve from FullRange multifocal Intraocular Lens, one year after surgery (n: 240 patients; defocus addition from +3.0 to -4.0 D). Abbreviations: n: number; D: diopter; LogMar: Logarithm of the Minimum Angle of Resolution.

The mean ± SD of preoperative SE was 2.0 ± 2.18 D (range: -5.50 to 4.75), which decreased 12 months after surgery to -0.04 ± 0.28 D (range: -0.75 to 0.625). The SE of post-operative refraction is illustrated in Figure 1, showing that most of eyes obtained SE values between -0.50 and 0.50 D. There was no loss of lines of vision and 98.3% of patients achieved UDVA between 20/20 and 20/25 (58.1% 20/20 and 40.2% 20/25), as can be seen in Figure 2. The UNVA (binocular) obtained was J1 for 72.5% and J2 for 27.5% of patients. All the patients were able to see the computer screen at 70 cm (arms’ length) one year after surgery (UIVA). Good outcomes were obtained for different defocus additions, as can be seen in Figure 3, with 0.04 logMAR for -3.0 D (near sight), 0.09 logMAR for -1.5 D (intermediate sight) and 0.03 logMAR for 0 D (distance sight). The best sight was 0.02 logMAR, achieved for 2.5 D of defocus. Table 1 shows the answers from satisfaction questionnaires, where most patients indicated that they obtained spectacle independence, achieving a high percentage of surgical expectations. Also, Halos were perceived in a low percentage of cases and only 1% said that it bothers all the time.

The mean ± SD of CCT was increased by 6.62 ± 2.79 micrometer (1.24%), the mean ± SD of ECD was decreased by 226.08 ± 11.63 cell/mm^2^ (9.00 %), both with statistically significance, and the IOP remained stable one year after surgery, without statistically significance, as shown in Table 2.

**Table 1:** Questions and Answers From the Satisfaction Questionnaires in 240 Patients One Year After FullRange Multifocal Intraocular Lens implantation.

Questions	Answers
Have you obtained spectacle independence?	92%, yes. 4%, need spectacles sometimes to read. 2%, need spectacles sometimes for driving/watching television. 2%, need spectacles sometimes for digital screens, not always.
Did the surgical outcome meet your preoperative expectation?	98%, yes. 2%, not at all.
Do you perceive “halos”? If yes, tell us if this bothers your visual activities, daily life or not (e.g. for night driving)	87%, no. 1%, yes, always, and they do bother my visual activities 5%, sometimes, and they do bother my visual activities. 7%, sometimes, but they don’t bother my life.

**Table 2:** Comparison of Mean Values, Standard Deviation (range) From Endothelial Cell Density (ECD), Central Corneal Thickness (CCT), and Intraocular Pressure (IOP) at Different Time-Points. The Statistically Significant Differences Were Compared (p< 0.05 in bold).

	Preoperative	6 months		12 months			p-value	
CCT (micrometers) Mean ± SD (range)	530.25±35.38 (435–642)		536.63±38.30 (445–680)	536.87±38.17 (445–687)	** 0.007**			
ECD (cell/mm^2^) Mean ± SD (range)	2511.12±213.64 (2023–3056)		2363.39± 197.10 (1897–2911)	2282.77±203.92 (1632–2891)	**0.00**			
IOP (mm of mercury) Mean ± SD (range)	**Preoperative**		**1 day**	**1 month**	**1 year**			
	13.98±1.78 (11–18)		13.88±1.79 (10–18)	13.98± 1.74 (11–18)	14.03± 1.72 (11–18)			0.62

## DISCUSSION

In the present study, one year after cataract surgery most patients obtained spectacle independence without noticeable complications. Their uncorrected near, intermediate and distance visual acuity were good enough, which meant that 98% of them achieved their preoperative surgical expectations. Furthermore, adverse visual effects, such as halos, occurred in a low percentage of cases (13%) and only 1% of them stated that it bothers continuously, affecting their lives. 

Every day surgeons (and patients) have more IOL models to choose for a cataract surgery. Which technique is the best for each patient depends on visual tasks, personal needs, budget, surgical technique and surgeon preference, as well as countries’ regulatory issues. Technical information provided by the commercial sponsors is not always easy to understand, with the laboratory and/or clinical information given not usually evaluated in “real practice”. Also, different biases could be presented which could influence medical decisions. Consequently, this study was designed in a practical manner to evaluate the clinical outcome of patients who underwent FullRange multifocal IOLs implantation, evaluating simple aspects of their visual function, surgical safety and patient satisfaction.

There are not many previously published scientific papers on the SeeLens MF platform. To the best of our knowledge, this study was the first regarding the FullRange multifocal IOL. An electronic search on this subject was performed in September 2019 on PubMed, PubMed Central and Google Scholar. In 2013, van der Linden et al [17] published a prospective study comparing the SeeLens MF IOL in 25 patients (48 eyes) with the SN6AD1 IOL (Alcon, Forth Worth, The USA) in 20 patients (37 eyes). Both are multifocal lenses, with near addition of +3.0 D in the IOL plane. In this work, after 3 months of follow-up, incidence of halos and distance and near visual acuities were similar, without statistically significant differences, but a clinically and statistically significant advantage was found for the SeeLens MF at distances of 50 to 60 cm. Also, straylight measurements were reported to be better for SeeLens. This aspect was later studied in more depth and published by Lapid-Gortzak et al [18], in a prospective cohort study, comparing both IOLs again (SeeLens vs SN6AD1). They found that the SeeLens MF IOL showed a straylight of log(s) 0.08 lower than the SN6AD1 IOL, 3 months after surgery, with similar results in terms of spherical equivalent and visual acuity. 

In the same year (2015) Alió et al. [19] published a study evaluating refractive outcomes and optical performance of SeeLens MF in 20 eyes with 6 months’ follow-up, when performing microincisional (MICS) surgeries. In addition, a control group of 21 eyes was used, where the monofocal Acrysof SA60AT was implanted. Alió et al. concluded that the MICS SeeLens MF IOL can restore distance and near vision in presbyopic patients after cataract surgery. The contrast sensitivity function (CSF) in photopic condition, and on the higher spatial frequencies, was within the physiological levels for the normal population of the same age group. Nevertheless, in scotopic condition, and on the remaining spatial frequencies, there was a reduction of the CSF after surgery. Montés-Micó et al. [24] previously reported that decrease in CSF in patients operated using multifocal IOLs is related to dispersed distribution of light within the optical surface, which is higher in low light conditions. This is similar to what Alió et al. [19] described in their SeeLens contrast sensitivity evaluation. The quality of the image in the retinal plane was improved, but was measured with a Hartmann-Shack aberrometer, and this wavefront technology has some limitations in evaluating the diffractive surface [25]. In the present work, quality of vision was not objectively measured, but it is an interesting aspect that should be evaluated in future.

The present study, performed with the FullRagne IOL had a longer follow-up (1 year) with more cases (480 surgeries), than the only 3 previous publishes studies, which evaluated the SeeLens MF IOL, from van der Linden et al. [17], Lapid-Gortzak et al. [18] and Alió et al. [19]. But aforementioned publications evaluated and objectively compared aspects of quality of vision between different multifocal IOLs, and the present work did not. Even that the aim and design of present study was different from those, visual performance achieved in all those evaluations were good enough at different distances (far, intermediate and near), as was also obtained in the present series. Those information, is associated to the “visual” efficacy, and quality of vision in this work was indirectly evaluated throughout the satisfaction questionnaire.

The subjective aspect of measuring the quality of life after surgery was evaluated in previous studies [24-26] by means of the VF-14 questionnaire [26, 27]. These studies showed high levels of patient satisfaction with performing their daily lives. In the present study, a very short questionnaire was used. And, even that we did not evaluate reliability and validity of the questionnaire (which is one limitation from this study), it was developed specifically to evaluate whether spectacle independence was obtained by patients, their preoperative expectations were achieved or if they suffer from perceive halos. The preoperative expectations were achieved in 98% of patients and 92% obtained spectacle independence. Halos were not noted by 87% of cases, and 7% of the remaining patients perceiving halos said that this does not affect their lives. Although the subjective evaluation about patient satisfaction developed in this study is short and rudimentary, the questionnaire represents a simple way to explore it.

Dissatisfaction after multifocal IOL implantation and the need to explain multifocal IOLs to patients has been extensively reported; dissatisfaction is principally associated with visual dysphotopsias (halos, starburst and glare) [28, 29]. The neuroadaptation “effect” after this kind of surgery is another relevant issue, and occurs fundamentally when clinical, refractive and/or quality of vision problems were not detected [30]. However, in medicine it is very important to establish a good doctor– patient relationship. This is a relevant issue which could influence patients’ satisfaction, but it could not be measured. In the present series, good SE refraction was achieved, the surgeries were performed without complications and posterior capsular opacification did not develop one year after surgery. These objective data support the good results observed in the defocus curve. However, whether it is enough to ensure the patients’ satisfaction is under question. The answer is possibly no, and patients’ satisfaction and their questionnaire answers are influenced by the surgeon’s attitude. Even though careful steps were taken to avoid bias and the “doctor’s influence on the patients’ answers” by the patients completing the questionnaire anonymously and at home, it is not possible to guarantee that totally. Potential influence of the surgeon at postoperative follow-up, his or her optimism or pessimism, is another interesting issue to evaluate in future studies. It is not enough to improve the optics of the eyes after ophthalmic surgeries. Moreover, the psyche of the patient could play a relevant role. Better knowledge about visual cortex plasticity [31, 32] associated with new technologies and discoveries about neuroadaptation, as in the work of Rosa et al [33], regarding neuronal changes observed by functional magnetic resonance imaging to assess patients with multifocal intraocular lenses, will open new ways to improved surgical results and patient satisfaction.

The objective information obtained in this study shows that better visual acuity (0.02 logMAR) was achieved at -2.5 D of defocus. The distance visual acuity (0 D of defocus) was also good (0.03 logMAR), showing two peaks of maximum vision, decreasing the acuity at intermediate vision (-1.5 D), but with very acceptable and useful sight (0.09 logMAR). The visual performance achieved in this study was better than that published by Alió et al. [19] and similar to the results presented by van der Linden et al. [17]. However, it is not possible to compare the present study with those studies because the surgical techniques, the number of patients and follow-up were different.

Some limitations were as follows; the present study was performed in only one center by only one surgeon and without a control group. The phacoemulsification surgical technique, avoiding the use of viscoelastic substance, should not affect the results, but understanding that it is still not a popular technique, the safety of this technique was evaluated. The difference in corneal parameters (ECD and CCT) one year after surgery was statistically significant, but was not clinically relevant and those values were similar (or better) than those observed after cataract surgeries performed by phacoemulsification or Femtosecond Laser-Assisted Cataract Surgery (FLACS) with viscoelastic substance [34, 35]. In the present work, the mean ECD decrease was 9.09 ± 8.93% 12 months after surgery; some studies have shown an ECD loss of 8.1 ± 8.1% 3 months after FLACS and a loss of 13.7 ± 8.4 % after standard phacoemulsification, without clinically significance for the corneal health [34, 35]. These data only demonstrate that the surgical technique without viscoelastic seems to be at least as successful as other techniques when viscoelastic substance is used. Nevertheless, it was not the purpose of this study to compare surgical techniques, and this aspect must be separately evaluated in future. Moreover, IOP remained stable, without a clinically and statistically significant difference.

Finally, the strength of this study was to be the first report with the largest series of surgeries implanting “FullRange” multifocal IOL with 1 year follow-up, evaluating visual performance obtained and patient´s satisfaction. It would be interesting to perform a future comparative study with objective measurements of quality of vision.

## CONCLUSION

In summary, the refractive efficacy of the FullRange multifocal IOL was proved in 480 eyes, and spectacle independence was achieved in 240 satisfied patients one year after surgery. No complications were detected and the posterior capsule remained clear one year postoperatively without the necessity for performing capsulotomy. A longer follow-up period is necessary to confirm the results from the present study. Further studies should include some evaluation about whether the surgeon’s attitude (preoperative and during follow-up) could influence the patients’ final satisfaction more than the refractive and visual results.
